# An Open-Source ROS-Gazebo Toolbox for Simulating Robots With Compliant Actuators

**DOI:** 10.3389/frobt.2021.713083

**Published:** 2021-08-11

**Authors:** Riccardo Mengacci, Grazia Zambella, Giorgio Grioli, Danilo Caporale, Manuel G. Catalano, Antonio Bicchi

**Affiliations:** ^1^ Research Center “Enrico Piaggio”, University of Pisa, Pisa, Italy; ^2^ Soft Robotics for Human Cooperation and Rehabilitation, Istituto Italiano di Tecnologia, Genova, Italy

**Keywords:** articulated soft robots, ROS-Gazebo simulators, compliant actuators, Sim2Real methods, digital twins

## Abstract

To enable the design of planning and control strategies in simulated environments before their direct application to the real robot, exploiting the *Sim2Real* practice, powerful and realistic dynamic simulation tools have been proposed, e.g., the ROS-Gazebo framework. However, the majority of such simulators do not account for some of the properties of recently developed advanced systems, e.g., dynamic elastic behaviors shown by all those robots that purposely incorporate compliant elements into their actuators, the so-called Articulated Soft Robots ASRs. This paper presents an open-source ROS-Gazebo toolbox for simulating ASRs equipped with the aforementioned types of compliant actuators. To achieve this result, the toolbox consists of two ROS-Gazebo modules: a plugin that implements the custom compliant characteristics of a given actuator and simulates the internal motor dynamics, and a Robotic Operation System (ROS) manager node used to organize and simplify the overall toolbox usage. The toolbox can implement different compliant joint structures to perform realistic and representative simulations of ASRs, also when they interact with the environment. The simulated ASRs can be also used to retrieve information about the physical behavior of the real system from its simulation, and to develop control policies that can be transferred back to the real world, leveraging the *Sim2Real* practice. To assess the versatility of the proposed plugin, we report simulations of different compliant actuators. Then, to show the reliability of the simulated results, we present experiments executed on two ASRs and compare the performance of the real hardware with the simulations. Finally, to validate the toolbox effectiveness for *Sim2Real* control design, we learn a control policy in simulation, then feed it to the real system in feed-forward comparing the results.

## 1 Introduction

The applications in which robots have to significantly interact with uncertain environments and to cooperate with the human are growing fast in the last years. One of the major contributions to this growth is given by the recent advances in robotics technologies which see the introduction of structures able to perform such ambitious tasks safely, e.g., by featuring compliance (Albu-Schaffer et al., 2008). There are multiple ways of embedding compliance into the system. One of the possible solutions to this aim is the use of compliant-actuated joints for the robotic system, which form the so-called Articulated Soft Robots (ASRs) (Della Santina et al., 2020). From one side, exploiting the insertion of a constant linear spring into the robot’s joint, which separates the actuation from the link. This solution is implemented in the Series Elastic Actuators (SEAs) (Pratt and Williamson, 1995). From the other side, more complex and non-linear compliant mechanisms which instead allow modulating the compliance at the joints, namely Variable Stiffness Actuators (VSAs) (Vanderborght et al., 2013), can be realized.

**FIGURE 1:**
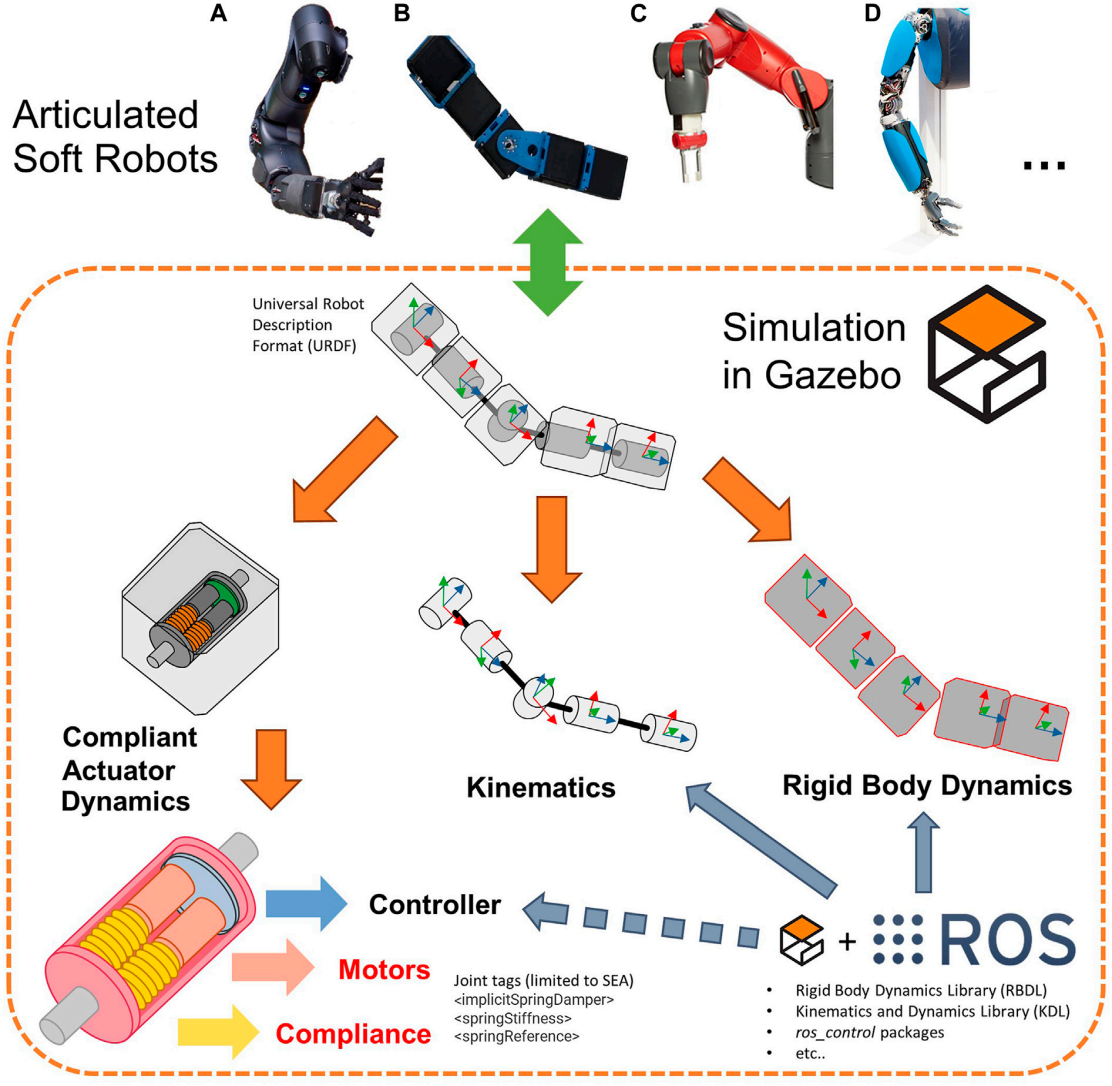
Available solutions for simulating in Gazebo the kinematics and the dynamics of ASRs, such as, **(A)** WalkMan’ arm (Negrello et al., 2015), **(B)** AlterEgo’s arm (Lentini et al., 2019), **(C)** Baxter’s arm (Guizzo and Ackerman, 2012), and **(D)** Hand-Arm system (Grebenstein et al., 2011). The tools for simulating the compliant-actuated and the motor dynamics are, however, limited to a few simple cases, i.e., the SEA ones. The proposed toolbox allows to reliably simulate also the dynamics of more complex ASRs.

Along with these technological advancements, the availability of effective dynamics simulators facilitated the development of more accurate control and planning strategies of ASRs. This, in turn, accelerated the process of designing and deploying complex compliant-actuated systems into real-world applications. A collection of such simulators is reported by Collins et al. (2021) in a recent survey paper and by Ivaldi et al. (2014). Although many of such simulators are mainly devoted to rigid-body systems, Collins et al. (2021) present also simulators specific for compliant (soft) robots. These simulators, however, do not consider the simplified case of robots equipped with compliant-actuated joints, but rather the case of systems with elastic bodies, i.e., continuum soft robots (Della Santina et al., 2020), which result even more challenging to be modeled and thus require ad-hoc tools. This class goes beyond the purposes of this work and, therefore, will not be covered. For the rigid-body class, instead, the most popular and widespread open-source dynamics simulators is Gazebo,^1^ according to Ivaldi et al. (2014), which offers an easy and intuitive connection with the Robotic Operation System.^2^ (ROS) middleware and the possibility of expanding its functionalities through the use of custom plugins (Cacace et al., 2020). Indeed, leveraging the ROS ecosystem, kinematics and dynamics libraries can be exploited to simulate real systems, as shown in Figure 1. Despite this, however, the possibility of simulating the complete dynamics of ASRs driven by compliant-actuated joints is not natively included within this powerful tool. In fact, only a few passive characteristics of the joints can be defined, such as the damping, the friction or the stiffness, and the equilibrium position, leveraging on specific URDF tags, while more complex passive mechanisms and possible dynamics of the motor actuation are not considered. The only way to implement such features is to leverage other software, e.g., Matlab/Simulink, or to use custom plugins. To the best of the authors’ knowledge, the only example of the latter solution has been presented in (Kameduła et al., 2016), where the authors developed a custom plugin specifically designed for simulating the SEA joints of a centaur-like platform. However, this solution has been only validated through comparative simulations in Matlab and is limited to the SEAs case, thus it neglects most of the other compliant platforms presented in the literature.

Motivated by this and by the need of validating in simulation controllers designed for compliant-actuated VSA structures, e.g., the one proposed in (Zambella et al., 2019), in this paper we present a ROS-Gazebo toolbox that allows simulating ASRs equipped with compliant joints in the class of SEAs and VSAs. The toolbox is composed of two main parts. The first one is used to generate a torque input to the rigid-body dynamics, leveraging the compliance model of the ASRs, and to simulate the dynamics of a direct current (dc) motor, which usually is considered as the actuation source of the compliant joints. The second part, instead, consists of a ROS node used to organize the output of the implementation and to achieve an easy-to-use interface for the overall toolbox. Furthermore, as an additional level of customization, the first part is implemented through a C++ class that can be derived from the users to implement different compliant joint characteristics, according to their platform.

Exploiting this toolbox, ASRs with compliant joints of different types can be accurately modeled and simulated in Gazebo. To this end, we release the implementation of some models relative to the compliant actuators presented by the VIACTORS.^3^ consortium, which started the investigation of variable stiffness technologies. The proposed toolbox can be easily incorporated in the Universal Robot Description Format (URDF) and, therefore, can serve to speed-up the design of other compliant actuators as well as collect useful and realistic simulation data for the development of planning and control strategies, e.g., for training and learning of specific tasks (Tutsoy et al., 2017). In this regard we show how, with this toolbox, it is possible to rely on simulation data, to design control policies for the real-world systems. As an example of this, we report the results of an Iterative Learning Control (ILC) procedure executed on a simulated platform, and then used to drive the real-world robot, achieving promising performance. In addition to this, simulations on the VIACTORS’s compliance models and experimental results on platforms equipped with VSAs are carried out to show the effectiveness of the toolbox and to show how the ASR behaves in simulation. The latter, indeed, we found to be closely comparable to the result obtained from the real applications, even in the case of an interaction scenario.

The paper is organized as follows. The dynamics of ASRs equipped with compliant actuators at the joints is given in Section 2, while the detailed description of the parts which compose the ROS-Gazebo toolbox is reported in Section 3. Simulation and experimental results, which validate and compare the outcome of the toolbox application with results on real platforms, are shown in Section 4. Conclusions are drawn in Section 5.

## 2 Articulated Soft Robots Dynamics

Leveraging the assumptions stated by Spong (1998), the dynamic model of a multiple degrees of freedom (DOFs) ASR equipped with compliant actuators at the joints can be described by the following contributions (Albu-Schäffer and Bicchi, 2016): the link-side dynamics, the motor-side dynamics and the compliance model that couples these two components.

### 2.1 Link-Side Dynamics

The link-side dynamics can be modeled as a rigid-body dynamics supposed driven by a torque source that, in case of ASRs, derives from the elastic mechanism of the compliant actuators. This dynamics is written as

$$M\left(q\right)\ddot{q}+C\left(q,\dot{q}\right)\dot{q}+G\left(q\right)=\tau_{\text{e},q}+\tau_{\text{ext}},$$ (1)

where $q\in R^{n}$ is the vector of link positions with its derivatives $\dot{q},\ddot{q}$, while $M\left(q\right),C\left(q,\dot{q}\right)\in R^{n\times n}$ and $G\left(q\right)\in R^{n}$ are the inertial, Coriolis/centrifugal, and gravitational terms of the system, respectively. The terms $\tau_{\text{e},q}\in R^{n}$ are the elastic torque components acting on the link, which depend on the compliance model of the system. Finally, $\tau_{\text{ext}}\in R^{n}$ are possible external torques.

### 2.2 Motor-Side Dynamics

The motor-side dynamics, instead, depends upon the motors which compose the compliant joints and is computed as

$$B\ddot{\theta }+D\dot{\theta }+\tau_{\text{e},\theta }=\tau_{\text{m}}-\tau_{\text{f}},$$ (2)

where $\theta \in R^{m}$ is the vector of motor positions with its derivatives $\dot{\theta },\ddot{\theta }$, and $B,D\in R^{m\times m}$ are the inertial and damping matrices of the motors, respectively. The term $\tau_{\text{m}}\in R^{m}$ is the vector of motor torque inputs, while $\tau_{\text{f}}\in R^{m}$ is the torque component due to the presence of friction. $\tau_{\text{e},\theta }\in R^{m}$ are the elastic torque components acting on the motors that depend, again, on the compliance model of the system, as detailed in the following. Furthermore, it is worth noting that most of the compliant actuators proposed in the literature are often equipped with an embedded low-level controller that is used to regulate the position of the motors. This basic control, indeed, allows simplifying the use of these devices that can be then regarded as servo-actuators. In this case, the motor torque input is computed e.g., as ${\bar{\tau }}_{\text{m}}\left(x,\hat{x}\right)$, where *x* is the state vector and $\hat{x}$ is the desired state vector used to close the control loop. Therefore, for this case, the motor dynamics can be neglected and the only contribution that drives the link-side dynamics will be the elastic torques.

### 2.3 Compliance Models

**FIGURE 2:**
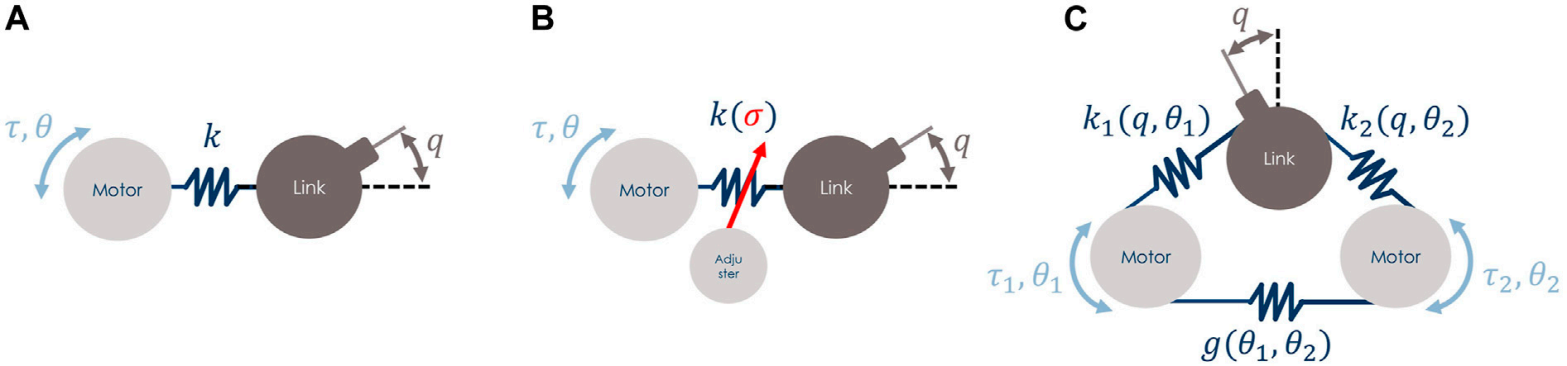
Examples of compliant actuator schemes that can be simulated with the proposed ROS-Gazebo toolbox, **(A)** is a SEA type, while **(B)** and **(C)** are VSAs. The latter two differ for the variable stiffness principle, **(B)** implements a VSA with Adjuster, while **(C)** shows a VSA with Agonistic-Antagonistic (A–A) motor-coupled principle.

Compliant actuators (Albu-Schaffer et al., 2008) consist of collocated motors *θ*, typically one or two, connected to the non-collocated link *q* *via* an elastic transmission mechanism, as shown in Figure 2. This implies that the terms *τ*_e,*q*_ and *τ*_e,*θ*_ in Eqs. 1, 2 are such that they couple these two dynamics components to form the complete dynamic model of ASRs.

According to the considered compliant actuator, the characteristics of its elastic mechanism imply different compliance models. As stated in Section 1, among the different types of compliant actuators, the most common are represented by the Series Elastic Actuators (SEAs) and the Variable Stiffness Actuators (VSAs) classes. Furthermore, to the best of the authors’ knowledge, almost all the VSAs presented in the literature are always composed of two collocated motors that are the minimum required to regulate either the link position and the joint stiffness. Therefore, the case of compliant actuators with more than two collocated motors will not be covered in this work.

The first class consists of an elastic element, i.e., a spring, interposed between the motor and the link, as shown in panel A of Figure 2. In this case, the number of motors of the system is equal to the number of links, i.e., *m* = *n*, and the spring is characterized by a linear and constant elasticity. The elastic torque is computed as

$$\tau_{\text{e},\theta }=k\left(q-\theta \right)=-\tau_{\text{e},q},$$ (3)

where the joint stiffness k∈R is positive and fixed and the difference *ϕ* = *q* −*θ* is named the deflection.

The second class, instead, is realized with more complex and non-linear compliant mechanisms through which the stiffness can be properly modulated. To allow stiffness modulation, the mechanism is equipped with an additional motor, thus the number of motors is twice the number of links, i.e., *m* = 2*n*. Two main implementation schemes exist in this case: 1) VSAs with Adjuster (panel B in Figure 2); 2) VSA with Agonistic-Antagonistic (A-A) principle (panel C in Figure 2). In the former scheme 1), while the first motor is elastically connected to the link, the second one is used to modulate the stiffness directly, typically mechanically, e.g., acting on a pivotal element. The dynamics of the second motor is usually faster than the one of the mechanical system, thus it can be neglected. This results in a kinematic control input for the stiffness variation, often denoted by the symbol *σ*. Consequently, the elastic torque term is computed as

$$\tau_{\text{e},\theta }=k\left(\sigma \right)f\left(q-\theta \right)=-\tau_{\text{e},q},$$ (4)

where the joint stiffness k(σ)∈R is still positive and can be modulated using the variable *σ*. Instead, the function f:R→R is typically chosen as a quadratic non-linear function in the deflection *ϕ*.

Differently, in the latter scheme 2), both motors are used to change the equilibrium position of the link as well as to modulate the joint stiffness, leveraging the agonistic-antagonistic principle. This principle is implemented by a carefully designed non-linear stiffness variation mechanism (Vanderborght et al., 2013). In this case, for each motor *i*, the elastic torque component is given by

$$\tau_{\text{e},\theta_{i}}=f_{i}\left(q,\theta_{i}\right),\;i=1,2,$$ (5)

where $f_{i}:R\to R$ is a non-linear function of, at least, class $C^{2}$. It is worth noting that here the elastic torque seen at the link, differently from the previous cases, is equal to

$$\tau_{\text{e},q}=\tau_{\text{e},\theta_{1}}+\tau_{\text{e},\theta_{2}},$$ (6)

that is a linear combination of the two elastic torque terms shown in Eq. 5. Furthermore, there might be cases for which the two motors have an additional elastic element between them, i.e., there is motor coupling (panel C in Figure 2). In this case, a further term *g*(*θ*_1_, *θ*_2_) must be added (or subtracted) in Eq. 5. In addition to this, for the VSA A-A case, leveraging the Equilibrium Point Hypothesis (EPH) state by Feldman (1986), a change of coordinate could be performed to control the two motor positions through two new variables *θ*_eq_, *θ*_sr_. These variables regulate the equilibrium position of the link and its stiffness preset, respectively, and the motor positions are such that *θ*_1_ = *θ*_eq_ + *θ*_sr_ and *θ*_2_ = *θ*_eq_ −*θ*_sr_. An example of this control solution is described in Mengacci et al. (2020).

## 3 ROS-Gazebo Toolbox Design

**FIGURE 3:**
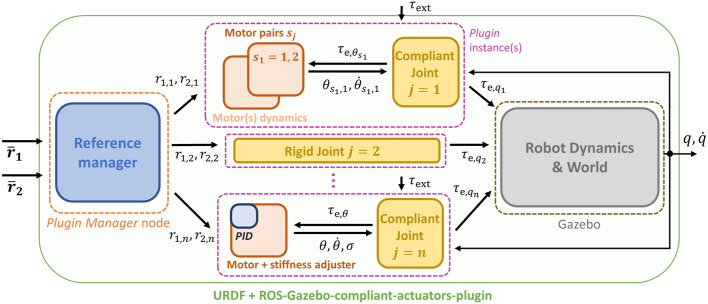
Scheme of the proposed toolbox integrated into the ROS-Gazebo framework, with main state and control variables exchanged between the components of the plugin and the node. Different compliant joints can be simulated as well as rigid ones, with and without motor dynamics and low-level PID control.

To simulate the complete ASR dynamics presented in the previous section, obtained by combining Eqs. 1, 2, we exploit the ROS middleware with the Gazebo simulation software. More specifically, we leverage the fact that Eq. 1 is commonly evaluated inside the Gazebo simulator with the support of open-source physic engines such as the Open Dynamic Engine.^4^ (ODE), starting from the robot’s model (i.e., a list of joints and links, with kinematic and dynamic parameters) described in the Universal Robotic Description Format (URDF) file. Regarding the elastic torque components *τ*_e,*q*_ and *τ*_e,*θ*_, the motor dynamics (Eq. 2), and the low-level controller, we developed a ROS-Gazebo toolbox which consists of two main parts: 1) 1 C++ plugin class in which the compliance model, as well as the motor dynamics and the controller, are implemented, and 2) a ROS node used to organize the input and output variables of the toolbox. In the following sections, we describe these parts in detail. The overall structure of the proposed toolbox is depicted in Figure 3.

### 3.1 Compliant Actuator Plugin

**FIGURE 4:**
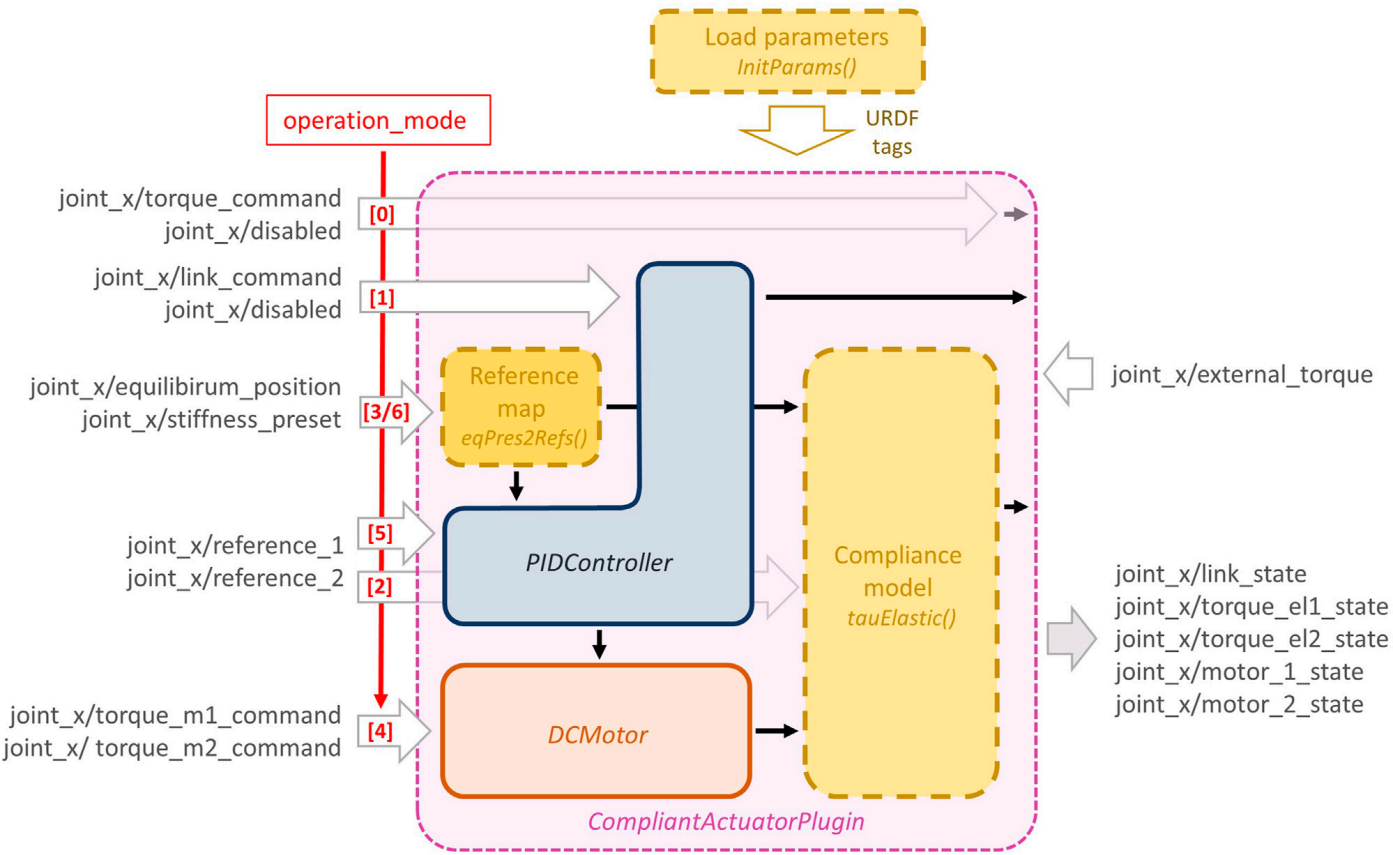
The detailed structure of the *CompliantActuatorPlugin*, with its components and the topic names with which it interacts. The dashed yellow boxes reported in this figure are *virtual* functions (see Section 3.1.3) of the main C++ class that can be customized and should be implemented by the user, while the other blocks are used to simulate the motor dynamics and a simple PID control loop.

**FIGURE 5:**
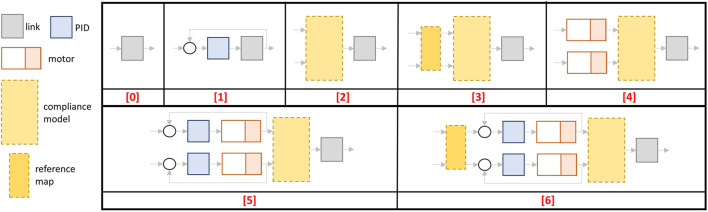
Control schemes of the operation modes implemented in the *CompliantActuatorPlugin*.

The purpose of this plugin, implemented as a C++ class, is to define how the elastic torque of the compliant actuator, i.e., τ_e,q_, is computed and to assign it to the joint torque of the Gazebo model. Furthermore, this plugin implements the dynamics of a direct current (dc) motor and a simple PID controller. The structure of this plugin, namely *CompliantActuatorPlugin*, is shown in Figure 4 and is composed of different blocks: 1) the compliance model, in which the elastic functions and their parameters [i.e., *f*, *f*_*i*_, *k*, *k*(*σ*) in Eqs. 3–5] are defined; 2) the *DCMotor* block, that implements the motor dynamics and from which the motor states, e.g., θ_1_, θ_2_, are retrieved; 3) the *PIDController* block, where the control law ${\bar{\tau }}_{\text{m}}\left(x,\hat{x}\right)$ is implemented, and 4) the reference map used to perform the change of variables from motor positions to equilibrium position and stiffness preset, i.e., θ_eq_, θ_sr_ or θ, *σ*. It is worth noting that points 1) and 4) depend on the specific compliant actuators that the user intends to simulate. For this reason, we implemented these two blocks as virtual functions of the main C++ class that the users can customize according to the compliance models of their platform. More details regarding this are given in Section 3.1.3, while points 2) and 3) will be described in Section 3.1.1 and Section 3.1.2, respectively. In order to simulate different control schemes that can be physically realized on the real compliant-actuated joints, we defined several operation modes, as shown in Figures 4, 5. Furthermore, to increase the general applicability of the toolbox, we leave also the possibility of simulating a rigid joint. Then, according to the operation mode selected by the user, the plugin class computes the torque for the joint, simulated in Gazebo, in different ways. Analogously, the topics from which the plugin retrieves the input commands, have different names. More in details, referring to Figure 4 and assuming that the simulated joint is named joint_x, the available operation modes and the relative command topics are given as follows:

0) this mode allows controlling directly the link torque *τ*_e,*q*_ (rigid actuation), passed from the topic named joint_x/torque_command. The other topic (joint_x/disabled) is not used;
1) differently from the previous operation mode, this one exploits the PID controller to regulate the link position *q* to the desired one, i.e., $\hat{q}$, passed from the topic named joint_x/link_command. Even in this case, the other topic (joint_x/disabled) is not used;
2) in this mode the input references, used to evaluate the elastic torque *τ*_e,*θ*_ or $\tau_{\text{e},\theta_{i}}$ from the compliance model, are passed through the topics joint_x/reference_1 and joint_x/reference_2;
3) here the motor positions for the compliance model are retrieved from the reference map. Thus the topics refer to the equilibrium position and the stiffness preset, passed through the topics named joint_x/equilibrium_position and joint_x/stiffness_preset, respectively;
4) this mode exploits also the dynamics of the motors. Thus the input references consist of the desired motor torques and are passed through the following topics joint_x/motor_1_command and joint_x/motor_2_command;
5) similarly to mode (2), here the topics are named joint_x/reference_1 and joint_x/reference_2, which, however, refer to the desired positions for the motors, regulated by the PID controllers;
6) as for the previous one, in this operation mode, the motor positions are regulated through the PID controllers, but the inputs refer again to the equilibrium position and stiffness preset variable, analogously to mode (3).

In addition to these topics, the plugin subscribes to a topic named joint_x/external_torque in which a possible external torque *τ*_ext_ can be defined. Furthermore, this plugin publishes the link position, velocity, stiffness, torque, and the value of the two references through the topic joint_x/link_state, while the state of the motors, where present, are published inside the topics named joint_x/motor_1_state and joint_x/motor_2_state. Finally, for the compliant-actuated joints cases, i.e., operation modes (2–6), the elastic torques are published as joint_x/torque_el1_state and joint_x/torque_el2_state.

### 3.1.1 DC Motor Dynamics Block

**FIGURE 6:**
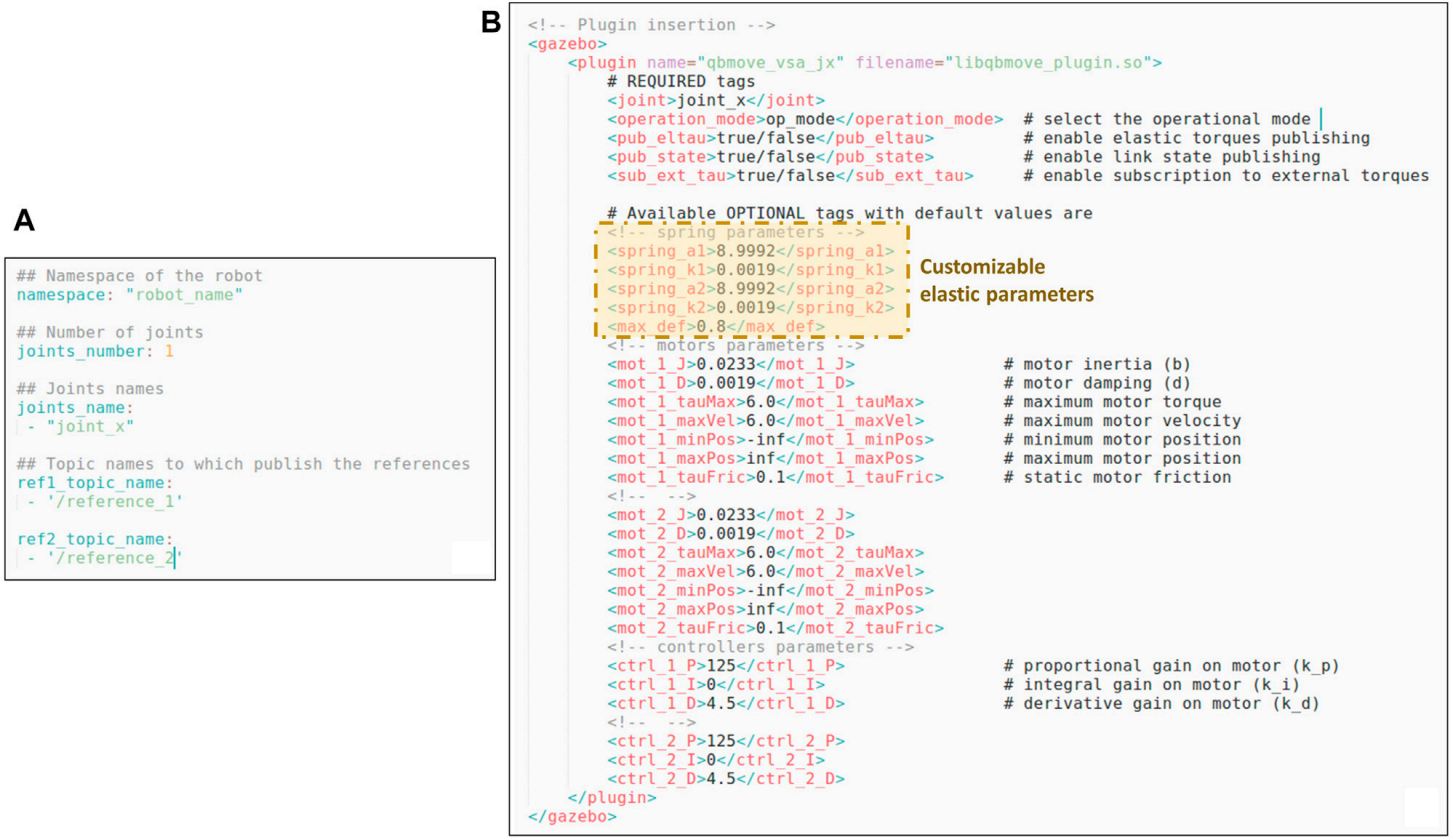
Panel **(A)** shows an example of the configuration file required by the *Plugin Manager* node, while in panel **(B)** the template for inserting the plugin relative to the qbMove Advanced discussed in Section 3.1.3, is reported. It is worth noting that, besides required tags for the plugin, there are also optional tags that the users can define to modify the motor and controller parameters. Furthermore, there are customizable parameters that depend on the elastic functions implemented in the compliance model.

In this block, referred to as *DCMotor* (orange box in Figure 4), the dynamics of a simple dc motor has been implemented, in order to compute the motor positions that are required from the compliance model in operation modes (4–6). We considered only the mechanical dynamics since the electric one is faster and, therefore, can be neglected. Thus, starting from Eq. 2, this block assumes the following dynamics for each motor *s*_*j*_ = {1, 2}

$${\ddot{\theta }}_{s_{j}}=\frac{1}{b}\left(u_{s_{j}}-d{\dot{\theta }}_{s_{j}}\right),$$ (7)

where b,d∈R are respectively the inertia and the damping constants of the motor (reflected at the link) and defined in the plugin insertion (Figure 6). The term $u_{s_{j}}\in R$ includes the *s*_*j*_-th component of the motor torque commands *τ*_m_, the contribution of the elastic torques *τ*_e,*θ*_ in Eq. 2, and a simplified component for the torque friction derived from the Dahl model (Dahl, 1968) and implemented as in (Hayward and Armstrong, 2000), i.e., *τ*_f_ = *K* (*θ* − *w*) in which *w* is evaluated with

$$w=\left\{\begin{array}{c} \theta +\frac{{\hat{\tau }}_{\text{f}}}{K},\;\text{if}\;\theta -w<-\frac{{\hat{\tau }}_{\text{f}}}{K}\;\\ \theta -\frac{{\hat{\tau }}_{\text{f}}}{K},\;\text{if}\;\theta -w>\frac{{\hat{\tau }}_{\text{f}}}{K}\; \end{array}\right.,$$ (8)

where *K* is the spring constant of the elastic model, while ${\hat{\tau }}_{\text{f}}$ is the amount of static friction torque to simulate on the joint. Despite its simplicity, this choice allows maintaining a passive implementation, independent from the sampling time adopted for the simulation. Hereinafter the subscript *s*_*j*_ will be omitted for the sake of clarity. To implement this dynamics in ROS, (Eq. 7) must be reformulated in state-space form as follows

$$\left[\begin{array}{c} x_{1}\\ x_{2} \end{array}\right]=\left[\begin{array}{cc} -\frac{d}{b} & 0\\ 1 & 0 \end{array}\right]\left[\begin{array}{c} x_{1}\\ x_{2} \end{array}\right]+\left[\begin{array}{c} \frac{1}{b}\\ 0 \end{array}\right]u,$$ (9)

where the state $x={\left[x_{1},\;x_{2}\right]}^{T}={\left[\theta^{˙},\;\theta \right]}^{T}$. Then (Eq. 9) should be discretized. Among the different methods (Quarteroni et al., 2010), we selected the forward Euler technique, for which $x_{i}\left(k\right)=T^{-1}\left(x_{i}\left(k\right)-x_{i}\left(k-1\right)\right),\;i=1,2$, where k∈Z is the discrete-time variable and T∈R is the discretization time. This choice allows us to maintain a simple and efficient implementation. Nevertheless, other different approaches can be tested to improve the accuracy of the discretization. By applying the Euler integration method, (Eq. 9) becomes

$$\left[\begin{array}{c} x_{1}\\ x_{2} \end{array}\right]=\left[\begin{array}{cc} 1-\frac{d}{b}T & 0\\ T & 0 \end{array}\right]\left[\begin{array}{c} x_{1}\left(k-1\right)\\ x_{2}\left(k-1\right) \end{array}\right]+\left[\begin{array}{c} \frac{T}{b}\\ 0 \end{array}\right]u\left(k\right).$$ (10)

### 3.1.2 PID Controller Block

In addition to the motor dynamics presented in Eq. 10, in the *CompliantActuatorPlugin* we also implemented the low-level position controller discussed in Section 2.2, used in operation modes (1), (5) and (6). This is done by implementing a simple PID controller of the following form

$${\bar{\tau }}_{\text{m}}=k_{\text{p}}e+k_{\text{i}}\int e\text{d}t+k_{\text{d}}\dot{x},$$ (11)

where $e=\hat{\theta }-\theta$ is the *s*_*j*_-th motor position error with its first derivative $\dot{e}$, while $\hat{\theta }$ is the desired motor position. $k_{\text{p}},k_{\text{i}},k_{\text{d}}\in R$ are the control gains of the PID, passed to the plugin as parameters. Eq. 11 is evaluated at each step inside the controller block named *PIDController* (blue box in Figure 4).

### 3.1.3 Customizable Functions

In order to realize other custom plugins exploiting the proposed toolbox, the users must derive the main class presented in Section 3.1 and insert their compliance model, as well as passing the model parameters and define the reference map. This can be achieved by implementing the following *virtual* functions of the main class (dashed yellow boxes in Figure 4).

**FIGURE 7:**
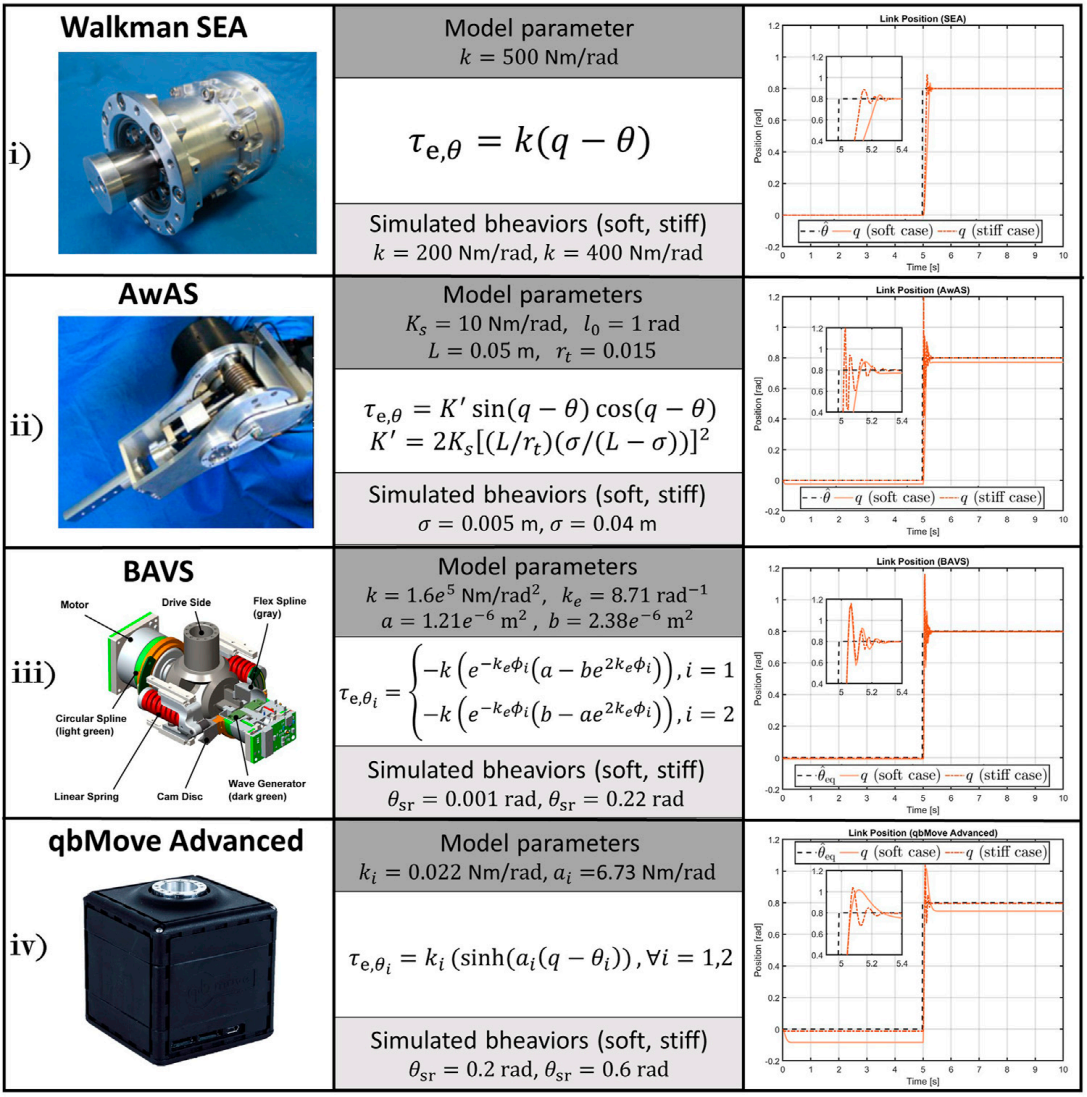
VIACTORS’s compliance models implemented in theROS-Gazebotoolbox and simulation results to the step response performed with different compliant behaviors (right-hand column). The 1-DOF structures simulate a mass of 0.2 kg attached to the output link, with the center of mass located at 0.04 m w.r.t. the joint axis, subjected to gravity.

• *tauElastic*(), this function is used to implement the compliance model, i.e., to compute the elastic torque components of the motors (*τ*_e,*θ*_ or $\tau_{\text{e},\theta_{i}}$) as well as the elastic torque for the joint *τ*_e,*q*_ and the stiffness value $\frac{\partial \tau_{\text{e},q}}{\partial q}$. The inputs required from this function are the motor *θ*_1_, *θ*_2_ and the link *q* positions;
• *eqPres2Refs*(), this function, instead, implements the change of variables, i.e., how the motor positions can be derived from the equilibrium and stiffness preset values. This depend on the type of compliant actuator to be simulated
• *InitParams*(), this last function allows the user to create and define some useful optional tags for the plugin (see panel B in Figure 6). For instance, referring to Figure 6, it is possible to see that there are other tags inserted, besides the motors and controllers ones. These optional tags allow setting *a*_1_, *a*_2_, *k*_1_, *k*_2_, and the maximum deflection, which are parameters of the specific compliance model to be simulated, i.e., the qbMove Advanced actuator discussed in the following (shown in panel (iv) of Figure 7).

## 3.2 Plugin Manager Node

**FIGURE 8:**
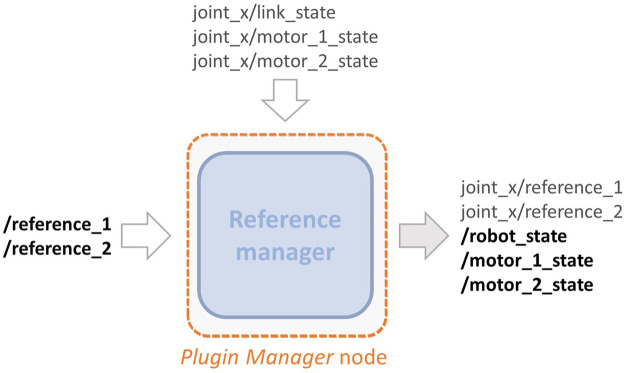
Detailed representation of the topics that the *Plugin Manager* node publishes and subscribes to.

This part consists of a ROS node that communicates with the plugin presented in the previous section, as shown in Figure 3. Given the modular structure of the blocks presented in Section 3.1, it results that a high number of topics are generated from the different joints. This fact, especially in the case of robots with a high number of joints, may increase the difficulty in passing the right commands to the relative joint and retrieving its information. For this reason, the goal of this node is to realize a more compact and clear implementation of the overall toolbox. To do so, according to Figure 8, this node acts in two ways: 1) allows passing the references to each joint starting from two simple and compact arrays; 2) subscribes to all the link and motor information published by the components of the plugin in order to collect them into arrays. To achieve the first objective, this node subscribes to two topics named / reference＿1 and / reference＿2, that are no more relative to the single joint, but to the whole robot model, referred to as robot＿name. In these two topics, there are arrays of the references, i.e., ${\bar{r}}_{1},{\bar{r}}_{2}$, passed by the user. Then, according to parameters retrieved from a configuration file in which the joint and reference names are specified (see panel A in Figure 6), it assigns these references to the relative joint.

Depending on the type of compliant actuator simulated and the operational mode selected (see Figure 4), the references will be different. More specifically we have that:

• for operation mode (0) the references is *r*_1,*j*_ = *τ*_e,*q*_, while for mode (1) is $r_{1,j}=\hat{q}$, the desired link position. In these modes the second reference is not assigned;
• in mode (2) the references are *r*_1,*j*_ = *θ*_1_ and *r*_2,*j*_ = *θ*_2_, while in mode (5) the references are the desired one $r_{1,j}={\hat{\theta }}_{1}$ and $r_{2,j}={\hat{\theta }}_{2}$;
• in case of operation modes (3) the references are *r*_1,*j*_ = *θ*_eq_ and *r*_2,*j*_ = *θ*_sr_, and for mode (6), these are the desired ones, i.e., $r_{1,j}={\hat{\theta }}_{\text{eq}}$ and $r_{2,j}={\hat{\theta }}_{\text{sr}}$;
• while for the remaining mode, i.e., (4), the reference are to the motor torque *r*_1,*j*_ = *τ*_m,1_ and *r*_2,*j*_ = *τ*_m,2_

The second objective of this node is achieved by subscribing to the following topics joint_x/link_state, joint_x/motor_1_state, and joint_x/motor_2_state of each joint that contain all the information relative to position, velocity, and torque of both link and motors, respectively, are published. Retrieved such information, the node copies those values inside three topics which deliver standard Joint State messages type. These topics are named / robot_state, / motor_1_state, and / motor_2_state and again refer no more to the single joint but to the entire robot model.

## 3.3 Examples of Derived Plugins

To show the versatility of the toolbox, and to simulate different platforms, we made available the implementation of some compliant actuators presented by the VIACTORS consortium. More in detail, referring to Figure 7, we implemented a simple SEA (e.g., the one presented in (Negrello et al., 2015)), the qbMove Advanced described in (Mengacci et al., 2021), the Bidirectional Antagonistic Variable Stiffness prototype (BAVS) presented in (Petit et al., 2015), and the Actuator with Adjustable Stiffness II (AwAS-II) proposed in (Jafari et al., 2011). These derived plugins are released within the proposed toolbox and are named, sea_plugin.cpp, qbmove_plugin.cpp, bavs_plugin.cpp, and awas_plugin.cpp, respectively. The compliance models of these actuators can be found on the datasheet of each prototype, according to the standard specifications presented by Grioli et al. (2015). Then, the users can leverage on these examples, as well as on a template one made available within the toolbox, to realize other plugins according to their compliant platform.

## 4 Toolbox Validation

**FIGURE 9:**
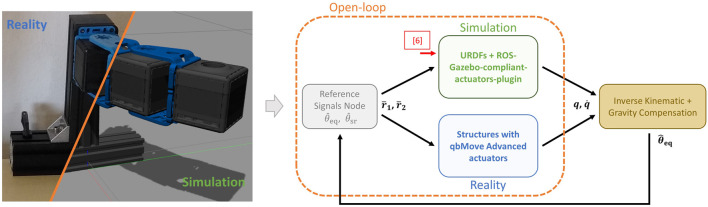
Control scheme used for both real experiment and simulated tests. Open-loop regulation (dashed orange box) has been exploited for the 1-DOF and 2-DOFs structures, while an inverse kinematic with gravity compensation approach has been used for the tests on the 5-DOFs arm.

To validate the performance of simulated ASRs realized with the proposed toolbox integrated with the Gazebo simulator, and to compare them w.r.t. the results obtained on the real-world platforms, we perform different simulation and experimental tests described in the following. More in detail, the first test reports the simulation of various compliance models, to show the capability of the proposed toolbox of simulating different compliant-actuated joints. Furthermore, a collection of experiments performed on real platforms.^5^ with multi-DOFs are presented to assess the reliability of the simulated counterparts, also in case of potential interactions with the external environment. The latter test is also useful to validate how the proposed toolbox reproduces the compliant behavior of the real platform. The control scheme used for these validation tests is reported in Figure 9. Starting from the encouraging validation results, then, we show how the proposed toolbox can reliably be used to transfer a control policy learned in simulation to the real-world platform, reducing the hardware time and leveraging the *Sim2Real* approach. Both simulations and experiments run on a desktop computer with a processor of Intel^®^ Core^TM^ i7-6700 CPU *@* 3.40GHz × 8, 16.5 GB RAM, equipped with Ubuntu 18.04.5 LTS, the ROS Melodic distribution, and Gazebo 9.

### 4.1 Simulated Compliant Actuator Models

At first, we simulated the different compliant actuators discussed in Section 3.1.3. As shown in Figure 7, we selected from VIACTORS’s website four actuators: 1) Walkman SEA, 2) AwAS II (VSA with Adjuster example), 3) BAVS, and 4) qbMove Advance (that are examples of VSA A-A case). Therefore, we considered four 1-DOF systems, each equipped with one of these compliant actuators and a mass of 0.2 kg attached to the output link at 0.04 m from the rotation axis of the joint, subjected to gravity. We analyzed the response of each system to a reference step of amplitude equal to 0.8rad. Since we do not know the mechanical parameters of the motors mounted on the different compliant actuators, we choose as operation mode the number (3). This allows us to control the joints directly through the compliance model inputs, according to the type of compliant joint. Therefore, an open-loop command is given as desired position $\hat{\theta }$ for 1) and 2) and as desired link equilibrium position ${\hat{\theta }}_{\text{eq}}$ for 3) and 4).

In addition to this, we considered two different compliant behaviors: *soft* and *stiff*. Referring to Figure 7, the elastic parameters that we took to simulate the soft behavior are, *k* = 200Nm/rad for the SEA actuator; *σ* = 0.005 m for the AwAS II; *θ*_sr_ = 0.001rad for the BAVS and *θ*_sr_ = 0.2rad for the qbMove Advanced, while for the stiff behavior, we considered *k* = 400Nm/rad, *σ* = 0.04m, *θ*_sr_ = 0.22rad, *θ*_sr_ = 0.6rad, respectively. The simulation results are reported in the right-hand column of Figure 7. From these plots, we can conclude that the toolbox is able to simulate different compliance models effectively. Furthermore, from the simulations, we can notice how also the compliant behavior is reproduced. This is particularly noticeable from the 2) and the 4) case, for which the link regulation, in the case of soft behavior, results affected by the gravitational contribution. Conversely, where stiffening the output link, the regulation is performed correctly.

### 4.2 Comparison of Simulations and Experiments

**FIGURE 10:**
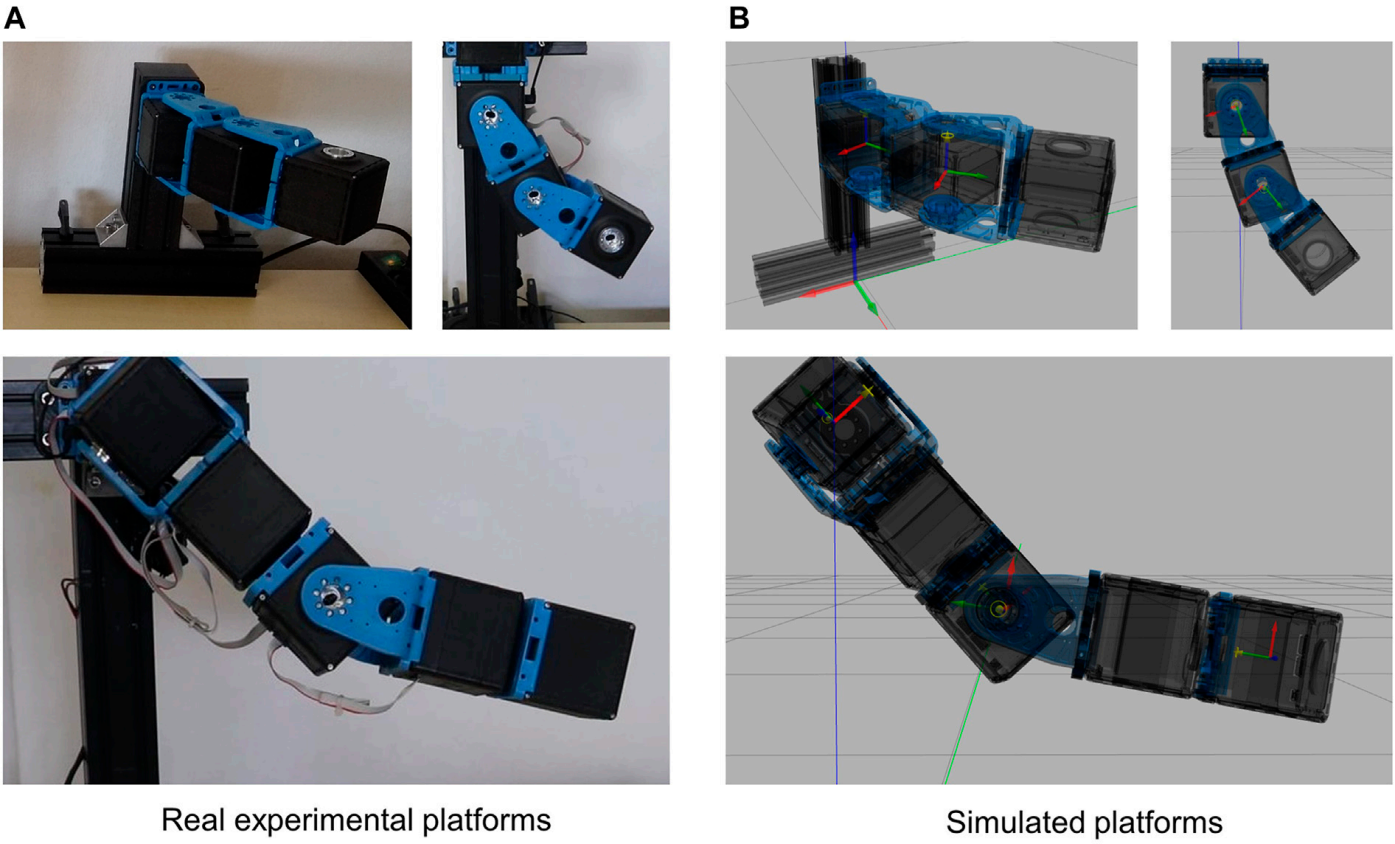
Structures used for the validation of the proposed ROS-Gazebo modules. The top figures in panel **(A)** and panel **(B)** show the 2-DOFs system in horizontal **(left-hand side)** and vertical **(right-hand side)** configuration, while the bottom figures depict the 5-DOFs experimental and simulated arm, respectively.

To assess the capability of the toolbox of replicating the performance of the real platforms in simulations, we carry out several experiments on different real and simulated platforms (Figure 10): 1-DOF and 2-DOFs systems, and a 5-DOFs arm, both composed of qbMove Advanced VSAs. More in detail, the 1-DOF is used to investigate the frequency response of the simulated compliant-actuated joint. Instead, we tested on the 2-DOFs system the capability of the presented toolbox of reproducing the actuator behavior when a trajectory is commanded as desired link equilibrium position ${\hat{\theta }}_{\text{eq}}$, with a fixed stiffness preset ${\hat{\theta }}_{\text{sr}}$ [operation mode (6)]. Furthermore, with the 5-DOFs robotic arm, we tested the stiffness reproduction when the system is interacting with the environment. Similar to the previous tests, for each experiment, we evaluated both the soft behavior and the stiff behavior, by changing the stiffness preset as *θ*_sr_ = 0.2rad and *θ*_sr_ = 0.6rad, respectively. We also supposed damping and static friction at the joints, defined from the URDF file as 0.065Nm/s and 0.025Nm, respectively.

These tests are described in the following (see also the Video attachment).

#### 4.2.1 Frequency Response

**FIGURE 11:**
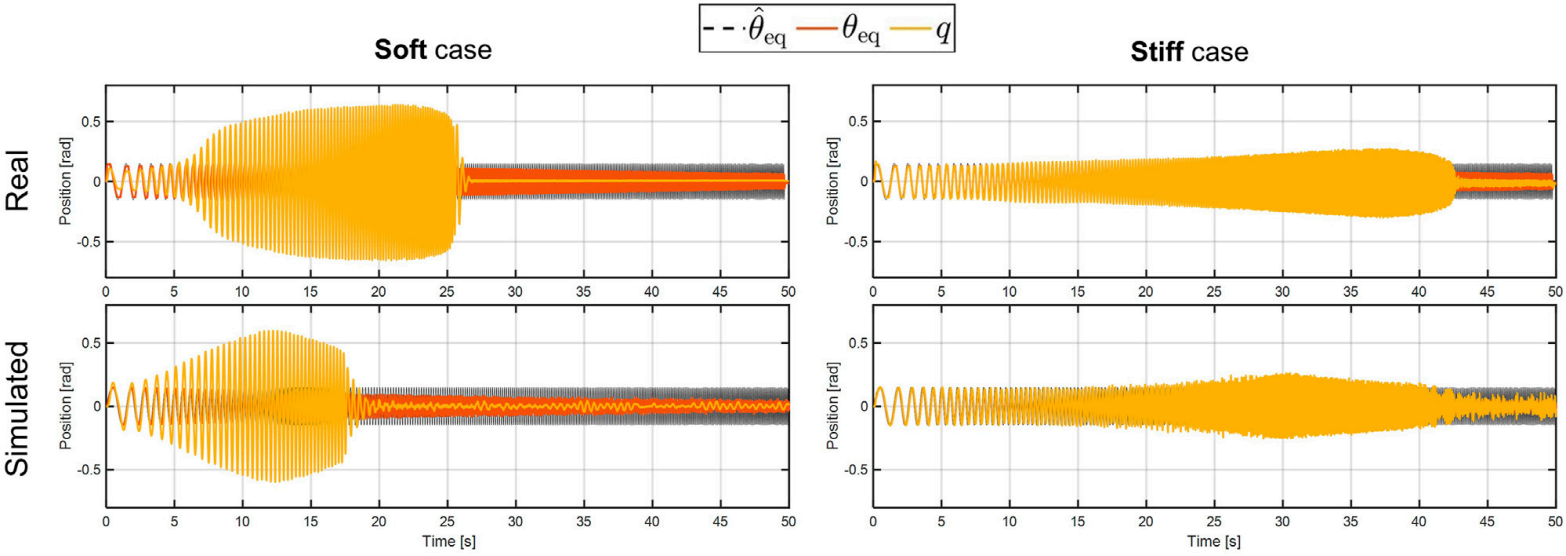
Experimental **(top plots)** and simulated **(bottom plots)** results of the frequency response test. The left-hand side shows the soft behavior, while the stiff behavior is reported on the right-hand side.

The first comparison is performed on the 1-DOF platform, subjected to a chirp signal of 0.15rad amplitude and ranging from 0.5 to 6 Hz of frequency. This test aims to evaluate the frequency response of the simulated compliant-actuated joint. As visible in Figure 11, the behavior of the simulated platform is comparable to the real one. More in detail, the value of the peak deflection reached by the link output is similar between the two cases, either in the soft and in the stiff behavior. Furthermore, the link equilibrium position of the simulated case (red lines in Figure 11) evolves as the real compliant actuator one. Despite this, however, there are few differences in the evolution of the output link and on the bandwidth of the two systems. These issues can be related to the joint parameters, i.e., the damping and the friction used by Gazebo to simulate the rigid-body dynamics, which have to be properly tuned in order to match the real performance. Moreover, non-linear unmodeled dynamics and simulation parameters, such as the sampling time, may affected these results. These facts will be better investigated in future works.

#### 4.2.2 Step Response and Trajectory Tracking

**FIGURE 12:**
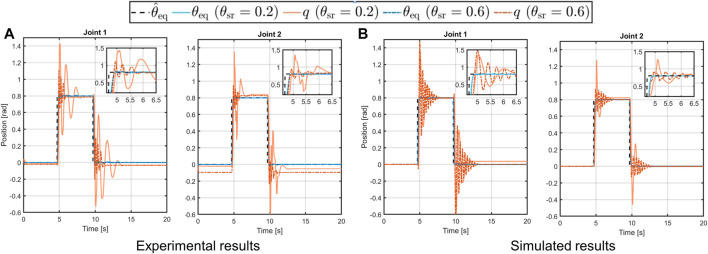
Experimental [panel **(A)**] and simulated [panel **(B)**] results of the 2-DOF structure in horizontal configuration following step references with soft (solid lines) and stiff (dashed-dotted lines) behavior.

As stated above, to show the capability of the toolbox to reproduce the real actuator behavior when a ${\hat{\theta }}_{\text{eq}}$ is commanded, we used the 2-DOFs platform. Thus, we apply to each joint of this system, mounted in a horizontal configuration (top-left picture in panel A of Figure 10), a step reference equal to the previous simulations. The experimental and simulated results are shown in panels A and B of Figure 12. Referring to this figure, we can see that the simulated behavior is closely comparable to the real one. In particular, we can conclude that either the link equilibrium position (solid and dashed-dotted blue lines) and the link output (solid orange line) achieve the desired position. However, it is worth noting that the simulated case in stiff behavior presents high oscillation phenomena w.r.t. the real case. This is due to the fact that in the real-world platform, according to Zhang et al. (2021), a change of the stiffness preset affects also non-linearly the damping seen at the link-side, while in the simulated case this parameter is kept fixed for both compliant behaviors.

**FIGURE 13:**
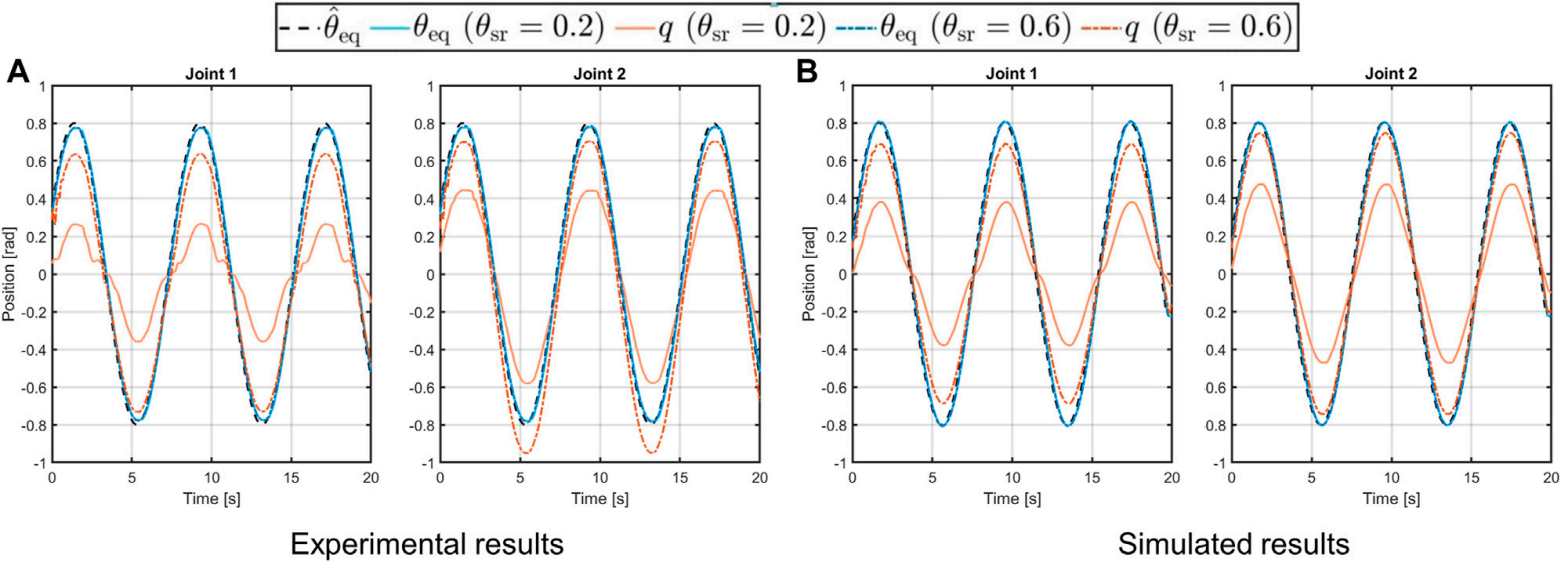
Experimental [panel **(A)**] and simulated [panel **(B)**] results of the 2-DOFs structure in vertical configuration performing a sinusoidal trajectory with soft (solid lines) and stiff (dashed-dotted lines) behavior.

The second test conducted on this platform consists of a trajectory tracking to a sinusoidal reference. Then, the desired trajectory is computed in open-loop with the following equation

$${\hat{\theta }}_{\text{eq}}\left(t\right)=H\sin\left(\omega t\right),$$ (12)

where the amplitude is chosen as *H* = 0.8rad, while the frequency is set as *ω* = 0.8 rad/s. In this case, the system is mounted in a vertical configuration, as shown in the top-right picture in panel A of Figure 10, thus it is subjected to gravity. From the results in Figure 13 we can see that, differently from the previous test, the simulated behavior of the link in the soft case (solid orange line), is not equal to the desired one, despite the link equilibrium position tracks the reference (solid and dashed-dotted blue line in Figure 13). This is due to the effect of the gravity torque on the compliance mechanism of the joint. Regarding the similarity of the simulated system w.r.t. the real platform, however, we can see that very similar behavior occurs also in the experimental platform, confirming that the toolbox is capable of reliably reproduce the real performance.

#### 4.2.3 Stiffness Reproduction During Interaction Tasks

**FIGURE 14:**
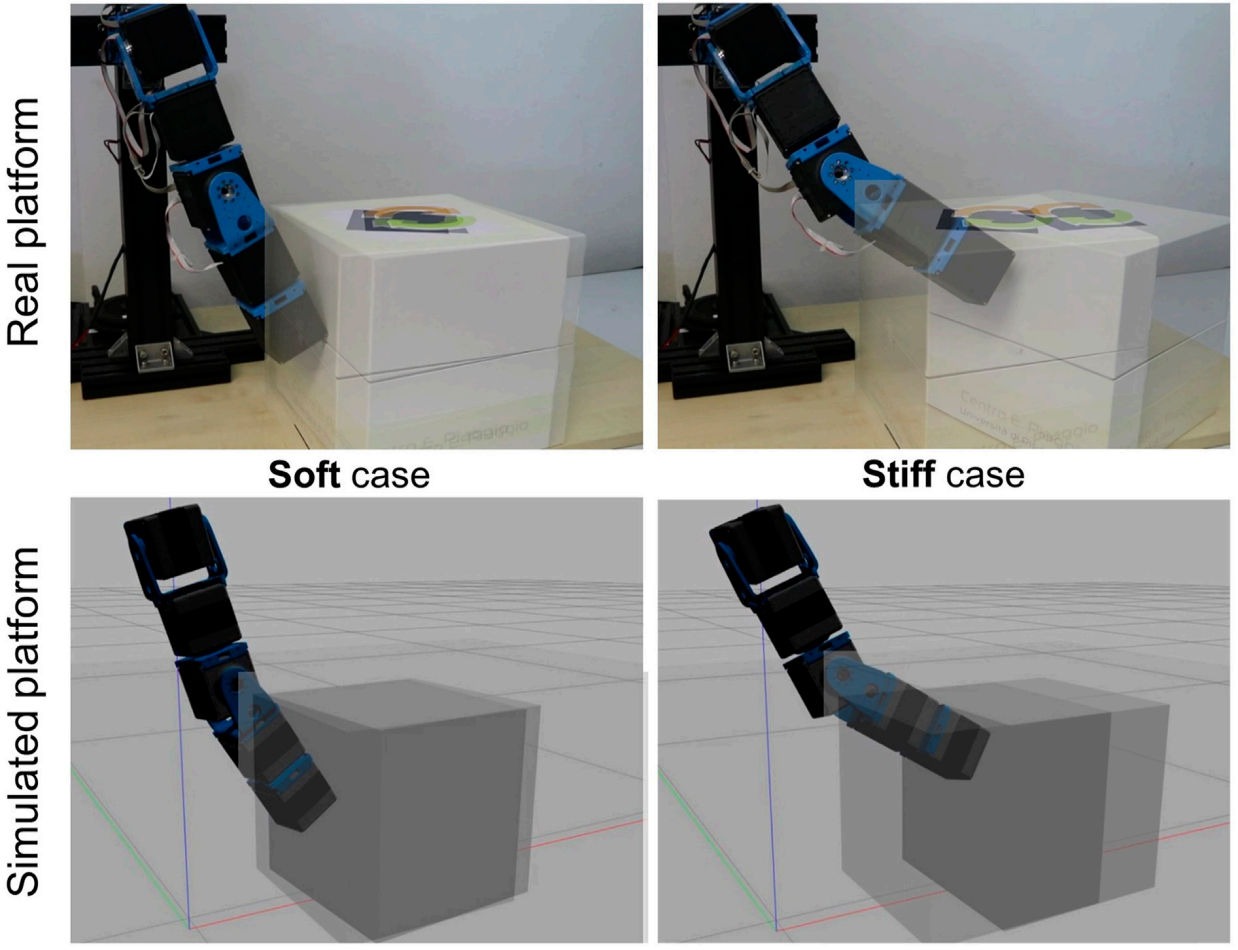
Experimental and simulated validations of the stiffness reproduction with the proposed ROS-Gazebo toolbox in case of an interaction task with a box of 2 kg weight. The right-hand side figures show the behavior in the soft scenario, while on the left-hand side the stiff case is reported. In the former case both the experimental and the simulated platforms are not capable to move the box, while if stiffened they can exert an increased force to the box able to move it forward.

In these experiments, we show the capability of the toolbox to reproduce the soft and stiff behavior of the system while interacting with the environment. In particular, we realized a 5-DOFs arm and commanded a desired trajectory to the end-effector. We implemented a first order closed-loop inverse kinematics (CLIK) (Siciliano et al., 2010) algorithm to find the corresponding desired link equilibrium positions ${\hat{\theta }}_{\text{eq}}$ for the different actuators. To compensate for the gravity effects on each joint, we computed and added to ${\hat{\theta }}_{\text{eq}}$ the deflection of the spring. Finally, we place a box of 2 kg weight and dimensions 0.265 × 0.265 × 0.265 m in the middle of the end-effector trajectory as an obstacle. For the simulated case, a wood-cardboard friction characteristics has been chosen for the contact between the box and the ground, by changing model parameters available in Gazebo.^6^ As illustrated in Figure 14, in the soft case, both in simulation and in the reality, the box stops the end-effector movement. Instead, in the stiff one, the arm moves the box to reach the desired end-effector position. These results, again, confirm that the compliant-actuated platform simulated with the proposed toolbox is able of capturing the behavior of the real ASR even in the case of non-negligible interactions with the external environment.

### 4.3 *Sim2Real* Example

**FIGURE 15:**
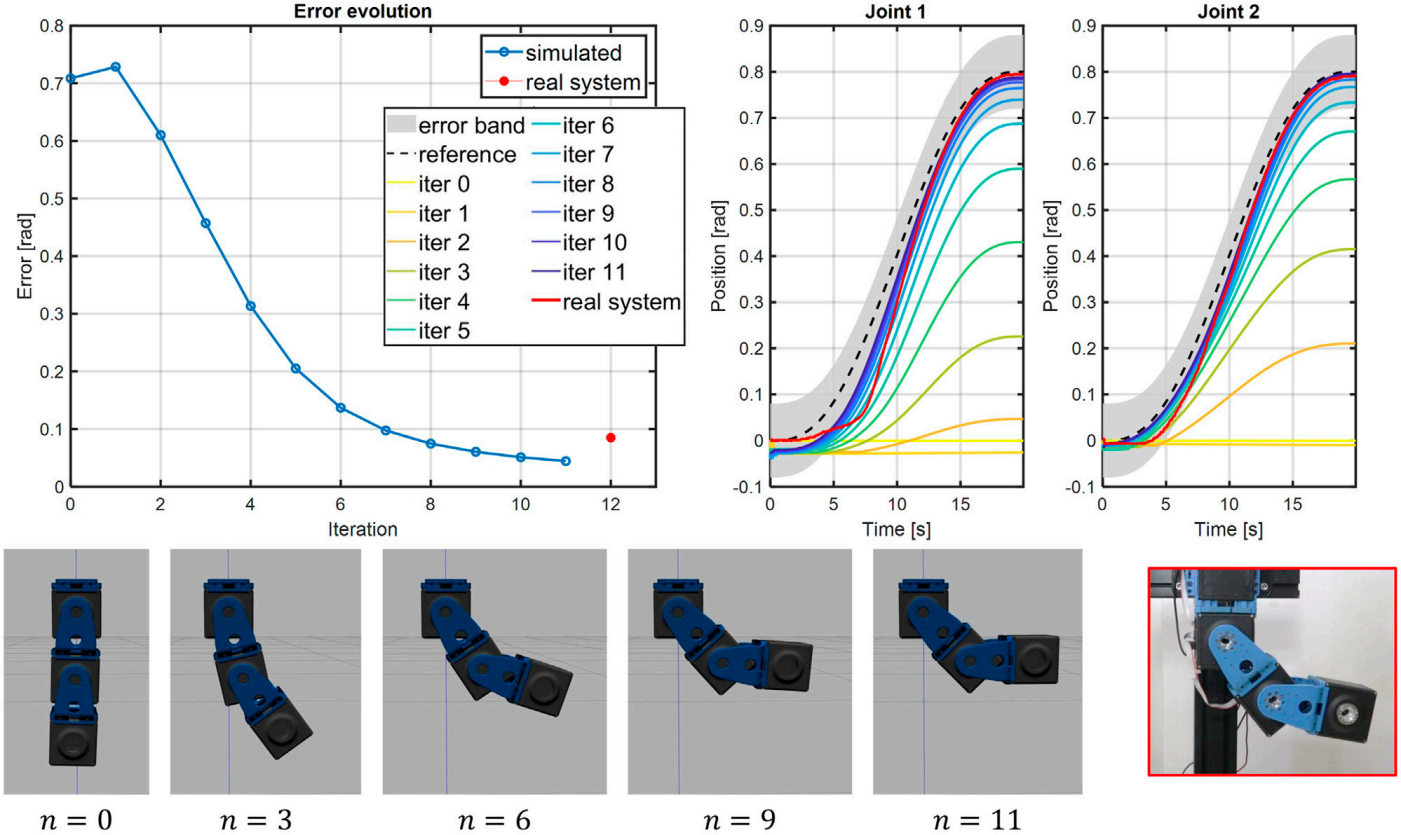
The Iterative Learning Control (ILC) procedure is executed on the simulated platform to retrieve the desired link equilibrium positions for the joints. Then, the control inputs are fed directly to the real system. Top-left plot shows the evolution of the error, computed as shown in Section 4.3, through the iterations, while top-right illustrates the trajectory tracking for each iterations and for the real test. The bottom figures show the frame sequences of the learning process and of the real hardware test.

To evaluate the real-world reliability of simulations executed with our toolbox, we performed a *Sim2Real* test in which a feed-forward control policy, learned iteratively in simulation, is used to drive a real-world structure. Iterative Learning Control (ILC) is a procedure through which one refines the time profile of the input that makes a robot execute a desired trajectory. We chose to test our simulations using the ILC paradigm for three different motivations. The first motivation is that ILC is recently being re-proposed as a promising technique to design the control of ASRs, and in particular those with variable stiffness (see, e.g., (Angelini et al., 2018)) since they do not alter the mechanical impedance characteristic of the ASR as feedback based controls do. The second motivation lies in the fact that ILC, being based on repeated iterations, highlights the usefulness of *Sim2Real* approaches in reducing the requirements in terms of hardware time. The third and final motivation is that the output of ILC is transferred to the hardware completely in feed-forward. This makes this test particularly challenging, since we avoid relying on the intrinsic robustness typical of feedback controller. We want to design the profile of the equilibrium position of the ASR shown in Figure 10 (top-right picture) to follow a fifth-order minimum-jerk trajectory that goes from zero to *q*_*i*_ = [0.8,0.8]^*T*^rad in 20s. The ASR is a 2-DOF arm, subject to gravity, actuated by two qbMove Advanced actuators (panel D in Figure 7). The parameters of the simulated compliance model were identified on the real platform using a procedure similar to that described in (Grioli et al., 2015). The resulting set is *k*_1_ = 6.257Nm/rad, *a*_1_ = 0.01038rad and *k*_2_ = 3.9Nm/rad, *a*_2_ = 0.08918rad for the first joint and *k*_1_ = 3.383Nm/rad, *a*_1_ = 0.1015rad and *k*_2_ = 7.011Nm/rad, *a*_2_ = 0.05944rad for the second joint. We set the value of the stiffness regulation parameter for both joints to *θ*_sr_ = 0.2rad, which corresponds to the soft case considered in the previous experiments. Then, we apply an ILC scheme similar to that presented in (Angelini et al., 2018). According to this ILC scheme, at each iteration n∈N the link equilibrium position for the ASR is updated according to the value of the link equilibrium *θ*_eq_ (*n* − 1) and the output link position error *e*_*q*_ (*n* − 1), computed at the iteration *n* − 1. The update law is

$$\theta_{\text{eq}}\left(n\right)=\theta_{\text{eq}}\left(n-1\right)+K_{\text{off}}e_{q}\left(n-1\right),$$ (13)

where *K*_off_ is the offline proportional gain, chosen iteration-dependent as *K*_*q*_ *e* ^−(*n*/10)^ with *K*_*q*_ = 0.5. We stop iterating once the maximum error on both joints is within a nominal tolerance band of 0.08rad. The ILC algorithm converges after 11 iterations, after which the learned input is fed to the real hardware platform and executed. The results of the ILC and of the hardware test are reported in Figure 15. As visible in the top-left plot in figure, the iterations performed in simulations allow the system to learn the control input, i.e., the link equilibrium position, required to perform the trajectory tracking. After that, the same control input is used to drive the real system, achieving a comparable error. For both cases the error is computed as $e=\|\hat{q}-q{\|}_{2}$. From the error plot, and from the trajectory tracking shown in the top-right corner, we can appreciate that the simulated and real platforms perform very similar. This can be also visually evaluated from the bottom plot in Figure 15, where frame sequences of the ILC process and of the subsequent test on the real system are shown (see also the Video attachment). Looking at the detail of the trajectories of the real robot, we can notice how the trajectory of the second joint is always within the nominal tolerance band of 0.08rad, and is also very close to the simulation. For the first joint, on the other hand, we see how the nominal tolerance band is exceeded for a small time between 5 and 9 s. We believe that this slightly inferior performance is due to the combination of two effects. The first is the notorious difficulty in modeling static friction phenomena, which are hard to identify and tend to be time-varying. This difficulty affects, partially, our model and we aim at improving it in the future. The second motivation is that the first joint is affected by a larger load that changes with the angle of the second joint. Therefore, the small deviations from the nominal position of the second joint contribute to move the first joint out of its trajectory even more. Nevertheless, recalling also the purely feed-forward nature of the hardware experiment, we consider the performance to be more than acceptable. Finally, we remark that the results on the hardware are obtained without any iteration on the hardware side, thanks to the 11 iterations on the simulation, saving at least 220 s of hardware time. Therefore, we can conclude that the proposed toolbox can be a useful tool to reliably speed-up the design and control of ASRs based on a *Sim2Real* approach.

## 5 Conclusion

This work proposes an open-source ROS-Gazebo toolbox useful to simulate the dynamics of ASRs driven by compliant-actuated joints. The main goal of the toolbox is to allow the user to reliably simulate different dynamic models for the compliant joint, with or without considering the motor dynamics and a possible low-level controller. To achieve this, the toolbox leverages the ROS and Gazebo frameworks and is structured in two parts: one devoted to the implementation of a Gazebo plugin, which can be derived from the user, where the compliance models and the actuation dynamics are implemented, and the other one consists of a ROS node used to organize the input/output variables of the toolbox, intending to realize an easy-to-use interface. Furthermore, as an additional contribution, the users can customize the first part of the toolbox, implementing the compliance characteristics of their ASR. In this regard, to simplify this operation, together with the toolbox we released the implementation of different compliant actuators proposed by the VIACTORS’s consortium. Simulations on these latter actuators, as well as several experiments on structures with multiple DOFs, equipped with VSAs at the joints, are carried out to validate the overall toolbox. These tests showed that the toolbox is able to effectively reproduce the performance of the real-world platforms and also to reliably simulate the compliant behavior of the ASR during interaction tasks with the environment. Motivated by these validation results, we also show how the toolbox, leveraging the *Sim2Real* approach, can be used to learn a control policy entirely in simulation, that can be then transferred directly to the real-world platform, maintaining satisfactory performance. Future works intend to improve the integration of the toolbox with existing ROS-Gazebo packages, e.g., the *ros_control* (Chitta et al., 2017), to realize a more general control toolbox also for the case of ASRs.

## Data Availability

The datasets presented in this study can be found in online repositories. The names of the repository/repositories can be found below: the open-source ROS-Gazebo toolbox presented in this article is part of the open-source Natural Machine Motion Initiative (NMMI, https://www.naturalmachinemotioninitiative.com/) and can be found at the following GitHub repository [ROS-Gazebo-compliant-actuators-plugin] [ROS-Gazebo-compliant-actuators-plugin], together with the URDF models of the platforms used for the validation. The toolbox is released under the BSD 3-Clause license.

## Footnotes

1 Gazebo: http://gazebosim.org/

2 ROS: https://www.ros.org/

3 VIACTORS: https://viactors.org/

4
https://www.ode.org/

5 The URDF models of the simulated platforms, with the same mechanical characteristics of the real structures, are available in the GitHub repository of the toolbox.

6 In particular, we imposed the tag of the surface friction equal to 0.23, which corresponds to the wood-cardboard friction coefficient available online.
